# Passive Stiffness of Left Ventricular Myocardial Tissue Is Reduced by Ovariectomy in a Post-menopause Mouse Model

**DOI:** 10.3389/fphys.2018.01545

**Published:** 2018-11-05

**Authors:** Núria Farré, Ignasi Jorba, Marta Torres, Bryan Falcones, Julio Martí-Almor, Ramon Farré, Isaac Almendros, Daniel Navajas

**Affiliations:** ^1^Heart Failure Unit, Department of Cardiology, Hospital del Mar, Barcelona, Spain; ^2^Heart Diseases Biomedical Research Group, IMIM (Hospital del Mar Medical Research Institute), Barcelona, Spain; ^3^Department of Medicine, Universitat Autònoma de Barcelona, Barcelona, Spain; ^4^Unitat Biofísica i Bioenginyeria, Facultat de Medicina, Universitat de Barcelona, Barcelona, Spain; ^5^Institute for Bioengineering of Catalonia – The Barcelona Institute of Science and Technology, Barcelona, Spain; ^6^Department of Pneumology, Hospital Clinic Barcelona, Barcelona, Spain; ^7^CIBER de Enfermedades Respiratorias, Madrid, Spain; ^8^Institut d’Investigacions Biomediques August Pi i Sunyer, Barcelona, Spain

**Keywords:** heart tissue, stress-strain, decellularized tissue, female hormones, ovariectomy

## Abstract

**Background:** Heart failure (HF) – a very prevalent disease with high morbidity and mortality – usually presents with diastolic dysfunction. Although post-menopause women are at increased risk of HF and diastolic dysfunction, poor attention has been paid to clinically and experimentally investigate this group of patients. Specifically, whether myocardial stiffness is affected by menopause is unknown.

**Aim:** To investigate whether loss of female sexual hormones modifies the Young’s modulus (E) of left ventricular (LV) myocardial tissue in a mouse model of menopause induced by ovariectomy (OVX).

**Methods:** After 6 months of bilateral OVX, eight mice were sacrificed, fresh LV myocardial strips were prepared (∼8 × 1 × 1 mm), and their passive stress–stretch relationship was measured. E was computed by exponential fitting of the stress–stretch relationship. Subsequently, to assess the relative role of cellular and extracellular matrix components in determining OVX-induced changes in E, the tissues strips were decellularized and subjected to the same stretching protocol to measure E. A control group of eight sham-OVX mice was simultaneously studied.

**Results:** E (kPa; m ± SE) in OVX mice was ∼twofold lower than in controls (11.7 ± 1.8 and 22.1 ± 4.4, respectively; *p* < 0.05). No significant difference between groups was found in E of the decellularized tissue (31.4 ± 12.05 and 40.9 ± 11.5, respectively; *p* = 0.58).

**Conclusion:** Loss of female sexual hormones in an OVX model induces a reduction in the passive stiffness of myocardial tissue, suggesting that active relaxation should play a counterbalancing role in diastolic dysfunction in post-menopausal women with HF.

## Introduction

Heart failure (HF) is a global health problem associated with high mortality and healthcare system expenditure (Farré et al., 2016, 2017). Whereas HF may occur in presence of either normal or abnormal left ventricular (LV) ejection fraction, diastolic dysfunction is common in almost all HF patients (Nagueh et al., 2016). Sex differences in prevalence and outcomes of diastolic dysfunction and HF have been reported (O’Meara et al., 2007; Meyer et al., 2013; Gori et al., 2014) and might be partially explained by withdrawal of estrogens at the time of menopause (Merz and Cheng, 2016) given that functional estrogen receptors reside in the myocardium (Mahmoodzadeh et al., 2006). Indeed, myocardium remodeling can result from pathways involving sex hormones and the natriuretic peptide system, which modulates diastolic dysfunction and HF (Hong et al., 1992; Maffei et al., 2001; Lam et al., 2011; Goncalves et al., 2017; Subramanya et al., 2018; Ying et al., 2018). However, comorbidities (Abramov et al., 2011; Paulus and Tschöpe, 2013) and age (Loffredo et al., 2014) also affect cardiac function and could explain the differences observed in several patient studies. Thus, the isolated effect resulting from loss of female sexual hormones on changes in left ventricle (LV) in patients remains poorly known.

Moreover, previous data from animal models of ovariectomy (OVX) also provided conflicting results. Some rodent studies reported an increase in ventricular fibrosis (Lestari et al., 2016; Wang et al., 2016), change in collagen expression (Voloshenyuk and Gardner, 2010), and changes in echocardiographic parameters suggestive of diastolic function (decrease in the early mitral annular velocity and increase in the early transmitral filling velocity-to-early mitral annular velocity) (Wang et al., 2016; Alencar et al., 2017) after OVX. However, other reports showed no differences in quantitative histological analysis of interstitial collagen deposition (Bupha-Intr et al., 2011; Mori et al., 2011; Almeida et al., 2014; Alencar et al., 2017) nor in heart function parameters (LV end-diastolic pressure, dP/dT, and E/A ratio) (Mori et al., 2011; Almeida et al., 2014) between OVX and intact animals.

Diastolic dysfunction – characterized by increased LV end-diastolic pressure – can be caused by either prolongation of active myocardial relaxation or increased myocardial passive stiffness (Zile et al., 2004; Rommel et al., 2016). Main determinants of passive stiffness in myocardium are extracellular matrix (ECM) and cardiomyocytes (Borlaug and Paulus, 2011). However, data available from human (Borbely et al., 2005) studies do not provide clear insight on whether female sexual hormones modulate the passive stiffness of LV myocardium. Unfortunately, animal research also provides non-conclusive results. Indeed, passive stiffness measured in cardiomyocytes (Kalász et al., 2014) and in trabecular bundles isolated from rats after 2 and 14 weeks after OVX, respectively, did not found significant differences with controls (Bupha-Intr et al., 2011; Kalász et al., 2014).

Given the clinical importance to further our understanding of the role of menopause in diastolic dysfunction, this study was focused on specifically assessing whether loss of female sexual hormones induces a change in the passive stiffness of LV myocardial tissue in a conventional mouse model of OVX. The passive elastance of myocardial strips from fresh native hearts was measured before and after decellularization to assess the differential contribution of both ECM and cellular components in total LV tissue stiffness in this menopause model.

## Materials and Methods

This investigation –approved by the Ethical Committee for Animal Research of the University of Barcelona – was carried out on 23 C57BL/6J female mice (12 weeks old) randomly distributed into two groups: OVX and sham-operated (SHAM) mice (*N* = 12 and *N* = 11, respectively). Conventional bilateral OVX was carried out under intraperitoneal ketamine/xylazine (5/10 mg/kg) anesthesia in OVX mice as described in detail elsewhere (Torres et al., 2014). Mice in SHAM group were subjected to the same surgical procedure with exception of ovary excision. After 6 months of OVX/SHAM surgery, mice were euthanized by exsanguination under intraperitoneal urethane anesthesia (1 g/kg). Their hearts and uteri were immediately excised and weighed, and hearts were subjected to LV myocardial sample preparation for further imaging and mechanical measurements both carried out by investigators who were unaware of the sample group (OVX vs. SHAM).

Myocardial fibrosis was assessed in four OVX and three SHAM mice by Picrosirius-red (ab150681, Abcam, United States) staining of interstitial collagen in paraffin-embedded 5-μ thick LV tissue sections, as previously described (Ramos et al., 2014). Images (40×) covering each whole LV sample (five to seven images in each animal) were taken (Olympus BX51 microscope and Olympus DP50 camera), and interstitial collagen fraction was quantified (Metamorph, Molecular Devices).

For mechanical measurements, hearts from eight OVX and eight SHAM mice were placed at room temperature in phosphate buffer saline (PBS) (Sigma) immediately after excision. LV wall was excised and a strip of ∼8 × 1 × 1 mm was cut with a scalpel along the long-axis direction and its mass (*M*) was measured. Resting length (*L*_0_) of the strip was measured, and its cross-sectional area (*A*) was computed as

(1) $$A=\frac{M}{\rho .L_{o}}$$

where ρ is tissue density (assumed to be 1 g/cm^3^). One end of the strip was glued with cyanoacrylate to a hook attached to the lever of a servocontrolled displacement actuator, which allowed us to stretch the strip and to measure the stretched length (*L*) and the applied force (*F*) (Aurora Scientific, 300C-LR). The other strip end was glued to a fixed hook (Supplementary Figure S1). This commercially available device (force resolution of 0.6 mN, length resolution of 1 μm) was previously used for similar applications (Farré et al., 2018; Perea-Gil et al., 2018). Measurements were performed inside a bath with PBS at 37°C to maintain physiological conditions. Stress (σ) applied to the strip was defined as

(2) $$\sigma \frac{F}{A}$$

Tissue stretch (λ) was defined as

(3) $$\lambda \frac{L}{L_{o}}$$

LV myocardial strips were initially pre-conditioned by applying cyclical stretch at a frequency of 0.2 Hz and maximum stretch of 50% for 10 cycles. *L*_0_ was measured again and 10 more cycles were acquired. Tissue stiffness was characterized by its Young’s modulus (E) defined as dσ/dλ. E was computed at 20% stretch (λ = 1.2) by exponentially fitting the stress–stretch (σ-λ) curve (MATLAB, The MathWorks).

After measuring E in fresh native samples, each strip was decellularized as described in detail elsewhere (Perea-Gil et al., 2015). Briefly, LV myocardial strips were immersed in a 1% sodium dodecyl sulfate (Sigma) solution for 48 h, followed by 1% Triton X-100 (Sigma) for 24 h, with solution replacement every 24 h and constant moderate stirring. Finally, strips were washed for 24 h using PBS. After decellularization, each strip was subjected to a stress–stretch measurement.

Group data were computed as mean ± SE. The OVX vs. SHAM differences in body, heart, and uterine weights, and E were assessed by *t*-tests. Mann–Whitney U tests were used for skewed data. A *p*-value < 0.05 was considered statistically significant.

## Results

**FIGURE 1:**
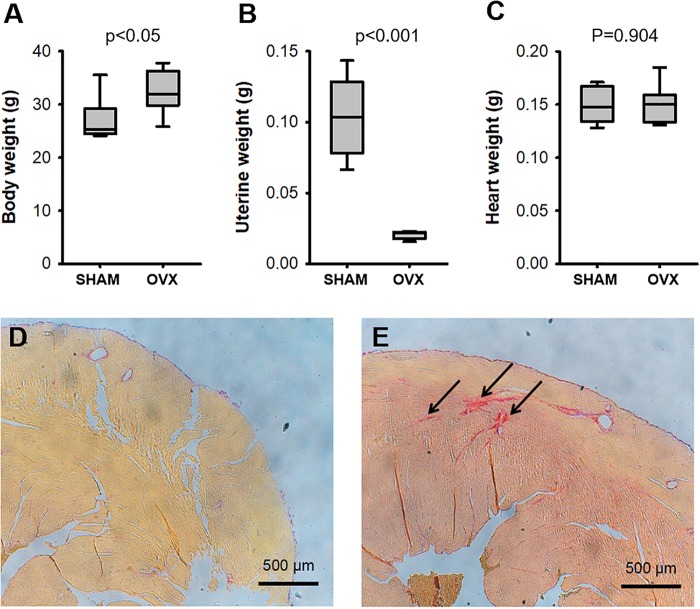
Body **(A)**, uterine **(B)**, and heart **(C)** weights in ovariectomized (OVX) and intact (SHAM) mice (*N* = 8). Picro-sirius staining (arrows) of the left ventricular myocardium in representative examples of intact **(D)** and ovariectomized **(E)** mice.

The OVX surgical procedure was effective as indicated by a considerable reduction in uterine weight and, as expected, OVX mice significantly increased body weight whereas heart weight did not change (Figure 1). Collagen fraction in LV myocardium (Figure 1) was significantly (*p* = 0.016) higher in OVX (2.04 ± 0.20) than in SHAM (1.00 ± 0.21).

**FIGURE 2:**
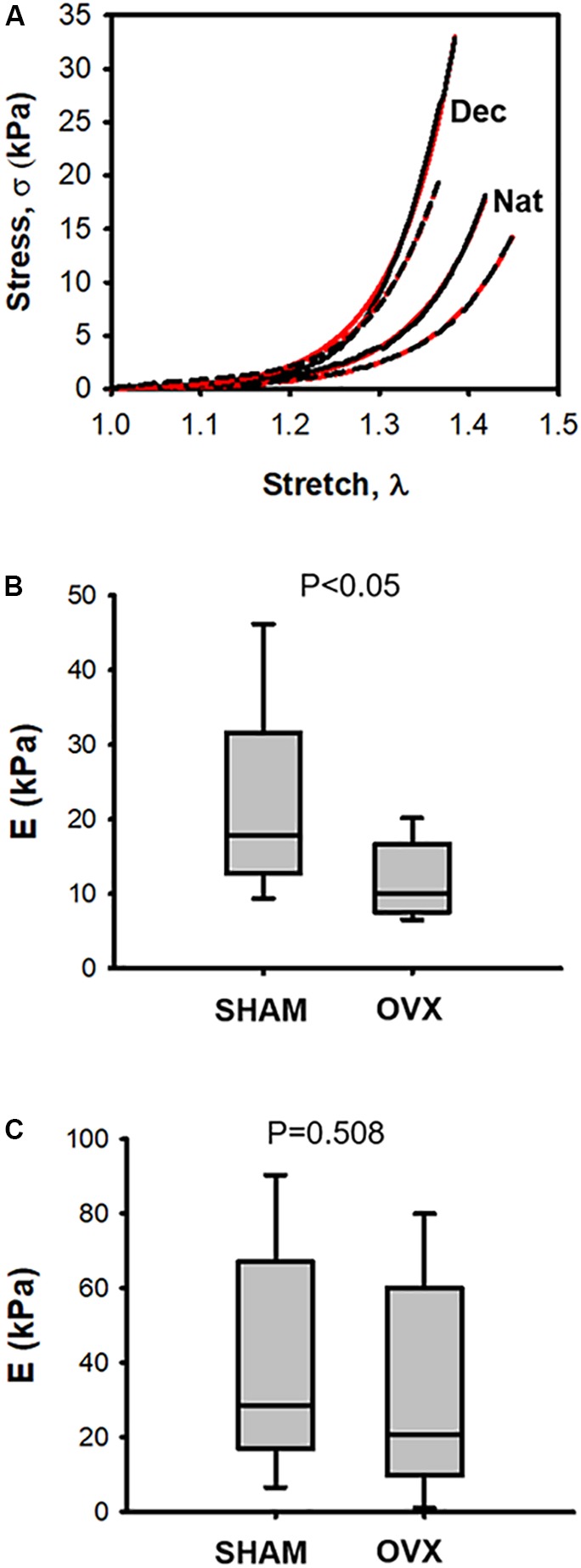
**(A)** Examples of stress–stretch curves in native (Nat) and decellularized (Dec) left ventricular myocardium tissue samples from one ovariectomized mouse (OVX; black dotted line) and from one intact mouse (SHAM; black solid line). Red solid and dashed lines correspond to exponential fits to the OVX and SHAM stress-stretch curves, respectively. **(B)** Passive Young’s modulus (E) of native left ventricular myocardium tissue computed at stretch λ = 1.2 in ovariectomized (OVX) and intact (SHAM) mice. **(C)** same as **(B)** in decellularized tissue (*N* = 8).

Representative stress–stretch curves in native and decellularized LV myocardium tissue samples for OVX and SHAM mice are shown in Figure 2A (raw data and exponential fit). Figure 2B shows that OVX reduced stiffness in fresh native LV myocardial tissue slices by ∼50% as compared with SHAM: *E* = 11.7 ± 1.8 kPa and 22.1 ± 4.4 kPa, respectively (*p* < 0.05). However, the stiffness of decellularized LV myocardial strips was not significantly different (*p* = 0.58) in OVX compared to SHAM: *E* = 31.4 ± 12.05 and 40.9 ± 11.5, respectively (Figure 2C).

## Discussion

This study shows that passive stiffness of fresh LV myocardial tissue was markedly reduced in ovariectomized mice when compared with same-age control animals. After tissue decellularization, no significant differences in ECM stiffness were found between both animal groups.

The rodent model of OVX is widely accepted in the literature to mimic menopause and hence to study the effects of lack of female sexual hormones on different organs and systems (Diaz Brinton, 2012). Contrary to studies in which animals were investigated a few weeks after OVX, we waited for 6 months after surgical intervention to avoid any risk of poor hormonal washout. Indeed, effectiveness of OVX was confirmed by marked ovarian atrophy as reflected by uterus weight (Figure 1).

To assess myocardial passive stiffness, we measured tissue mechanics of fresh LV myocardial strips. This approach allowed us to directly characterize tissue stiffness thereby avoiding the geometric assumptions required when tissue mechanics is determined from pressure–volume measurements in the whole ventricular cavity (Voorhees and Han, 2015). Tissue elasticity E was measured by slow strain-stress (0.2 Hz; a frequency lower than mouse heart beating) to reduce the well-known effect of strain rate on stress–strain relationship (Collins and Hu, 1972) and viscous components from the experimental setting. E was computed at 20% stretch since this is the range experienced by LV wall under physiological heart functioning conditions (Park et al., 2016). Stress–strain testing is a sensitive technique to detect changes in tissue stiffness, as it has been recently shown when using the same methodology to reveal that intermittent hypoxia mimicking sleep apnea increased LV ventricular ECM stiffness in mice (Farré et al., 2018). We measured the mechanics of fresh LV strips which mainly preserve the structure and composition of the native myocardial tissue (including the intact configuration of cells and ECM) and found a marked decrease in passive stiffness 6 months after OVX. However, it should be mentioned that LV myocardial strips do not exactly mimic the physiological *in vivo* configuration because of their limited size and lack of curvature. Therefore, future multiscale studies correlating the changes found at tissue level from strip measurements and alterations observed at physiological level *in vivo* (ventricular pressure–volume relationship) are warranted. A non-significant change in passive stiffness was observed in decellularized LV myocardial tissue. Noteworthy, the decellularized tissue was stiffer than the fresh native tissue (Figure 2), which could be a result of compaction of the acellular ECM architecture after tissue decellularization from recently excised fresh tissue (Andreu et al., 2014).

Loss of female sexual hormones has been considered a possible cause of ECM fibrosis, and hence of potential increase in passive stiffness, by accumulation and remodeling of collagen. However, the evidence available is conflicting since both increase and no change in collagen deposits after OVX have been reported (Voloshenyuk and Gardner, 2010; Bupha-Intr et al., 2011; Mori et al., 2011; Almeida et al., 2014; Lestari et al., 2016; Wang et al., 2016; Alencar et al., 2017). Although statistically significant, the augmented collagen deposition we found in OVX did not result in an apparent increase in myocardial tissue stiffness. The different changes observed in E before and after decellularization strongly suggest that cardiomyocytes play a major role in determining the changes in LV passive stiffness observed in native tissue after OVX. In fact, myocardial active relaxation should play a counterbalancing role in diastolic dysfunction (Kass et al., 2004; Zile et al., 2004), consistently with the fact that patients with HF and normal ejection fraction have significant abnormalities in both active relaxation and passive stiffness (Zile et al., 2004; Rommel et al., 2016). Although intermediate filaments could play a potential role, their contribution in OVX-induced changes are expected to be negligible given their small contribution in total passive cell stiffness (Kass et al., 2004). Consequently, most observed changes could be attributed to titin since this intracellular protein functions as a molecular spring that develops passive tension during diastole (Hutchinson et al., 2015; Voorhees and Han, 2015) and has been identified as a major determinant of cardiomyocyte passive stiffness (Borlaug and Paulus, 2011). However, studies that have analyzed the effect of OVX on titin have shown no significant differences in titin phosphorylation in isolated single cardiomyocytes (Kalász et al., 2014) or in the N2BA-to-N2B titin ratio in myocardium fibers (Bupha-Intr et al., 2011).

## Conclusion

Although elderly women are more likely to develop pronounced diastolic dysfunction than men (O’Meara et al., 2007; Gori et al., 2014), this study shows that OVX in mice was associated with a reduction in passive stiffness of fresh myocardial tissue. Our data unravel the role that female sexual hormones may play in modulating myocardial passive mechanics and raises the need for more detailed research on mechanisms involved in the clinically relevant question of post-menopausal diastolic dysfunction, in particular to investigate how the natural process of aging modulates the effect of loss of female sexual hormones.

## Ethics Statement

The experimental procedures were approved by the Ethics Committee of Animal Experimentation of the University of Barcelona following the local and European regulations in force.

## Author Contributions

NF and DN conceived the work and designed the experiments. IJ, MT, and BF carried out most experiments and data analysis. JM-A, RF, and IA contributed to analyze and interpret the data. All authors discussed the work and finally contributed to writing the manuscript final version drafted by NF and DN.

## Conflict of Interest Statement

The authors declare that the research was conducted in the absence of any commercial or financial relationships that could be construed as a potential conflict of interest.
