# Supplementary figures and images for: Passive Stiffness of Left Ventricular Myocardial Tissue Is Reduced by Ovariectomy in a Post-menopause Mouse Model
Source: Front Physiol. 2018 Nov 5;9:1545. doi: 10.3389/fphys.2018.01545 (PMC6230582; doi:10.3389/fphys.2018.01545)

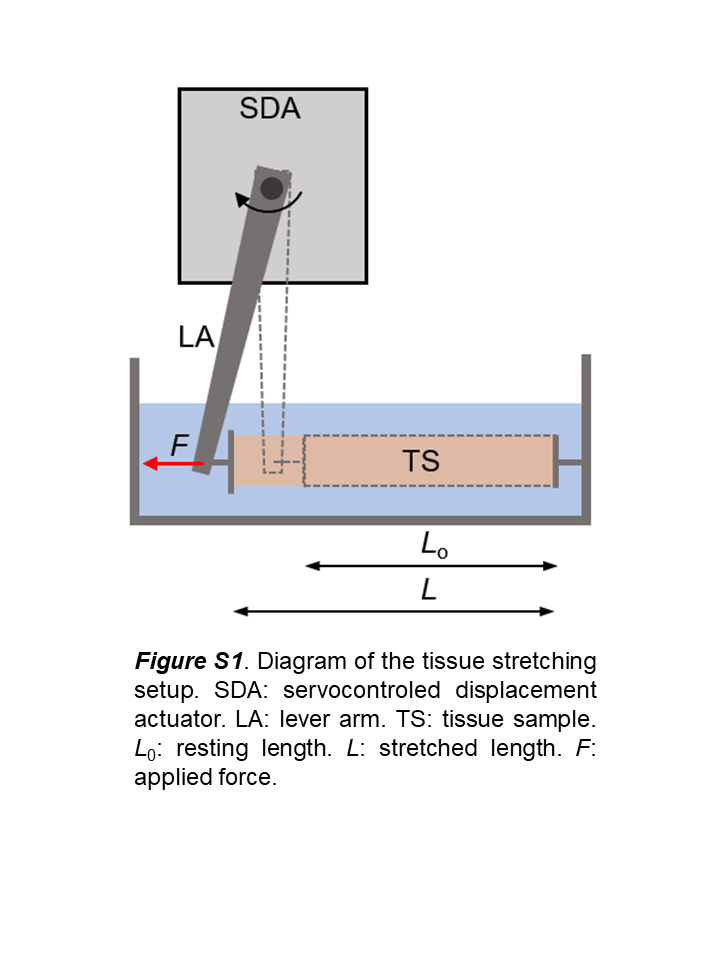

Supplement: Supplementary file 1 [file Image_1.TIF]
